# Effects of the new generation α-pyrrolidinophenones on spontaneous locomotor activities in mice, and on extracellular dopamine and serotonin levels in the mouse striatum

**DOI:** 10.1007/s11419-018-0409-x

**Published:** 2018-02-26

**Authors:** Jakub Wojcieszak, Dariusz Andrzejczak, Adam Wojtas, Krystyna Gołembiowska, Jolanta B. Zawilska

**Affiliations:** 1 Department of Pharmacodynamics, Medical University of Łódź, 1 Muszyńskiego, 90-151 Łódź, Poland; 2 Department of Pharmacology, Institute of Pharmacology, Polish Academy of Sciences, 12 Smętna, 31-343 Kraków, Poland

**Keywords:** α-PVP, PV8 and PV9, Spontaneous locomotor activity, Dopamine, Serotonin, Microdialysis, Synthetic cathinones

## Abstract

**Purpose:**

Pyrovalerone derivatives (α-pyrrolidinophenones) form a distinct branch of synthetic cathinones, a popular group of novel psychoactive substances, and exert strong psychostimulatory effects resulting from their high potency to inhibit dopamine (DA) and norepinephrine transporters, with negligible activity at the serotonin (5-HT) transporter. In contrast to the old generation α-pyrrolidinophenones, 3,4-MDPV and α-PVP, there is limited data on the pharmacology and toxicology of the novel analogs. Therefore, the present study assesses the in vivo effects of two new pyrovalerones, PV8 and PV9, along with those of α-PVP, on spontaneous locomotor activities of mice and extracellular DA and 5-HT levels in the mouse striatum.

**Methods:**

Spontaneous locomotor activity was measured using Opto-Varimex Auto-Track. Effects of tested compounds on extracellular levels of DA and 5-HT in the striatum were studied by an in vivo microdialysis technique; their concentrations in dialysate fractions were analyzed by high-performance liquid chromatography with electrochemical detection.

**Results:**

α-PVP, PV8 and PV9 stimulated mice locomotor activity (an effect being blocked by D_1_-dopamine receptor antagonist, SCH 23390), and increased extracellular levels of DA and 5-HT in the striatum. Observed effects depend on dose, time and compound under investigation, with α-PVP being more potent than PV8 and PV9. When used at the same dose, the pyrovalerones produced effects significantly weaker than a model, old generation psychostimulant, methamphetamine.

**Conclusions:**

Enhancement of dopaminergic neurotransmission plays a dominant role in the psychomotor stimulation caused by α-PVP, PV8 and PV9. Extending an aliphatic side chain beyond a certain point leads to the decrease in their potency in vivo.

**Electronic supplementary material:**

The online version of this article (10.1007/s11419-018-0409-x) contains supplementary material, which is available to authorized users.

## Introduction

Among recreational drug users, there has been a significant increase in the use of novel psychoactive substances (NPS) in recent years. Drugs belonging to one of the most prevalent groups of NPS are synthetic cathinones endowed with psychostimulatory action, and synthetic cannabinoids [1]. Synthetic cathinones emerged on the drug market in 2004 and since that time their number has been steadily increasing. To date, 118 synthetic cathinones have been detected. In 2015, these drugs constituted one-third of the total NPS seizures in the European Union, Norway and Turkey [1, 2]. Pyrovalerone derivatives (α-pyrrolidinophenones) form a distinct branch of synthetic cathinones. A key feature of the chemical structure of α-pyrrolidinophenones is the replacement of the primary or *N*-methyl amine with a pyrrolidine ring, and an extension of the α-carbon side chain. The most prominent member of the first generation of pyrovalerones is 3,4-methylenedioxypyrovalerone (3,4-MDPV), detected for the first time in Japan in 2007 [2, 3]. Following the scheduling of 3,4-MDPV in many countries, its further derivatives began to appear on the “recreational” drug market. The deletion of a 3,4-methylenedioxy group gave rise to α-pyrrolidinopentiophenone (α-PVP; “flakka”), which has been present on the European drug market since at least 2011 [4], and is one of the most prominent second generation pyrovalerones. Further modification of the α-carbon side chain length resulted in the introduction of new compounds, such as α-pyrrolidinobutiophenone (α-PBP), α-pyrrolidinopropiophenone (α-PPP), α-pyrrolidinohexanophenone (α-PHP; PV7), α-pyrrolidinoheptanophenone (α-PHPP; PV8), and α-pyrrolidinooctanophenone (α-POP; PV9) [2]. The latest two compounds were detected for the first time in Japan in samples collected in 2013 [5, 6].

Despite their relatively short presence on the clandestine market, α-PVP, PV8 and PV9, along with their phenyl ring-substituted derivatives, have been responsible for numerous cases of acute poisonings and fatal overdoses. Acute intoxication with these compounds can produce a wide range of symptoms, including sympathomimetic toxidrome (tachycardia, hypertension, agitation, increased aggression, chest pain, cardiac arrest), liver failure, psychiatric disturbances (paranoid psychosis, hallucinations, panic attacks, and suicidal ideations), seizures and acid-base imbalance [2, 4, 7]. By the middle of 2015, α-PVP alone was responsible for 105 fatal intoxications in Europe [4]. There are also reports of deaths from Japan, where PV8, PV9 and their substituted analogues were detected in biological samples taken postmortem [5, 8, 9].

Pyrovalerones exert strong psychostimulatory action, resulting from their high potency to block dopamine (DA) and norepinephrine (NE) transporters (DAT and NET, respectively), but lack empathogenic properties due to the negligible activity at the serotonin (5-HT) transporter (SERT). In contrast to several designer cathinones, α-pyrrolidinophenones act only as monoamine reuptake inhibitors. They do not enhance the release of neurotransmitters into the synaptic cleft. The potency of 3,4-MDPV and α-PVP to block DA reuptake, with IC_50_ values below 50 nM, is higher by at least one order of magnitude than that reported for methamphetamine and non-pyrovalerone cathinones [2, 10, 11]. It has been demonstrated that α-carbon side chain length is the key factor determining the affinity and uptake inhibition potency for DAT and NET, while the replacement of the pyrrolidine ring results in a loss of activity [12, 13]. Recent structure-activity studies show that, starting from α-PVP, subtraction of each carbon atom from the side chain results in a reduced potential to block DAT, but the extension of the side chain into PV7 and PV8 does not negatively affect the ability to inhibit DAT [10, 12]. Moreover, it is proposed that the ability to block DAT should improve with increasing bulk/lipophilicity of the side chain [13]. However, in contrast, anecdotal information obtained from web sites and forums for NPS users suggests that PV8 and PV9 are generally considered weaker psychostimulants than 3,4-MDPV and α-PVP; therefore, the suggested doses of PV8 and PV9 are 3–10 times higher than those of α-PVP [14–17].

**Fig. 1:**
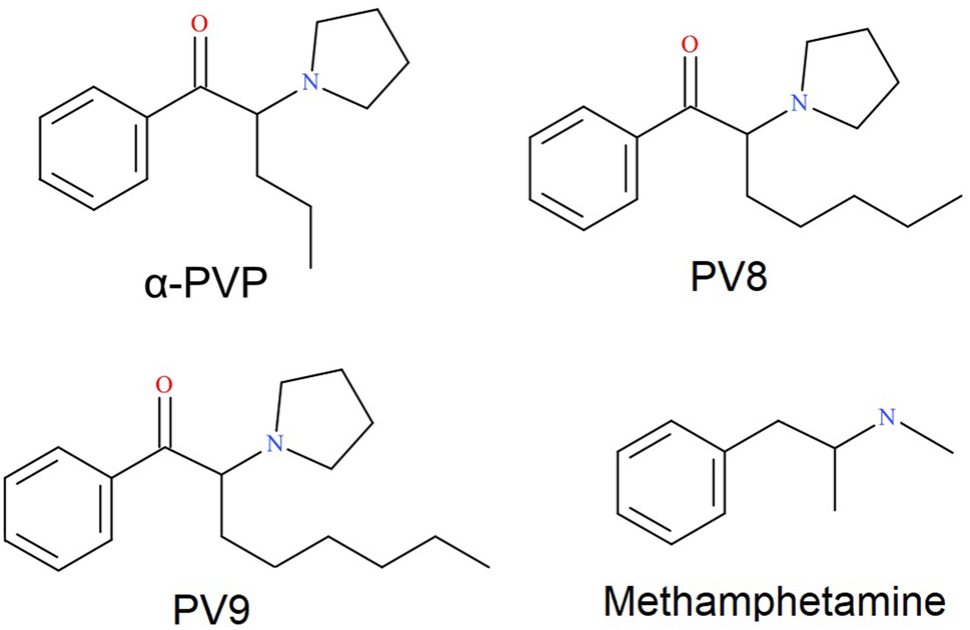
Chemical structures of psychoactive substances dealt with in this study

Although the pharmacological activity of α-PVP has been examined [10, 12, 18–21], to our knowledge there is only one paper on PV8 [12], and none on PV9. Therefore, the aim of the present work was to examine the effects of two scarcely studied pyrovalerones, PV8 and PV9, on spontaneous locomotor activity in mice, a widely-used behavioral test utilized to measure drug-induced psychomotor stimulation [18, 19]. The study also compares these results with the action of α-PVP, and that of methamphetamine, a classical, non-cathinone psychostimulant used as a reference compound (for chemical structures see Fig. 1). In order to determine whether the studied effects involve dopaminergic neurotransmission, two sets of experiments were performed. The first examined the effects of SCH 23390, a selective D_1_-DA receptor antagonist, on changes in mouse locomotor activity. The second used microdialysis to assess the drug-induced changes in extracellular DA levels in the ventral striata of freely-moving mice, and compared them with alterations of 5-HT.

## Materials and methods

### Reagents

Synthetic cathinones: α-pyrrolidinopentiophenone [α-PVP, 1-phenyl-2-(pyrrolidin-1-yl)pentan-1-one], α-pyrrolidinoheptanophenone [PV8, 1-phenyl-2-(pyrrolidin-1-yl)heptan-1-one], α-pyrrolidinooctanophenone [PV9, 1-phenyl-2-(pyrrolidin-1-yl)octan-1-one], and methamphetamine were purchased in the form of their hydrochloride salts from Cayman Chemical (Ann Arbor, MI, USA). Isotonic saline for injections (0.9% NaCl) was purchased from Polska Grupa Farmaceutyczna (Łódź, Poland). D_1_-DA receptor antagonist SCH 23390 (8-chloro-2,3,4,5-tetrahydro-3-methyl-5*R*-phenyl-1*H*-3-benzazepin-7-ol) hydrochloride was purchased from Sigma-Aldrich (Poznań, Poland). The chemicals used for high-performance liquid chromatography (HPLC) were obtained from Merck (Warsaw, Poland). Ketamine hydrochloride and xylazine were purchased from Biowet (Puławy, Poland).

### Animals

Experiments were performed on adult male C57BL/6J inbred mice at approximately 12 weeks of age. The animals were housed four per cage, under a 12-h light/12-h dark cycle (lights on at 06:00 a.m.), with free access to standard food and tap water. Experiments were conducted during daytime (08:00–14:00). All housing conditions and procedures were in accordance with the European Union guidelines regarding the care and use of laboratory animals (Council Directive 86/609/EEC of November 24, 1986).

### Locomotor activity

The study was conducted using Opto-Varimex Auto-Track (model 0271-002M, Columbus Instruments, Columbus, OH, USA) open field locomotor activity measuring chambers (20.3 × 20.3 × 20.3 cm) housed within sound-attenuating chambers in sets of four. A panel of infrared beams (16 beams) and corresponding photodetectors, spaced by 1.3 cm, were located on the *X* and *Y* horizontal axes. Additionally, identical sets of infrared emitters and detectors were installed on the higher layer in order to detect vertical movements. Experiments were conducted in a sound-attenuated room lit with a dim red light from above.

Tested compounds were dissolved in 0.9% saline and injected subcutaneously (s.c.) in a volume of 100 μL/10 g body weight. Separate groups of eight mice were injected with either vehicle (0.9% saline) or α-PVP (1, 3, or 10 mg/kg), PV8 (3, 10, or 15 mg/kg), PV9 (3, 10, or 15 mg/kg), or methamphetamine (0.3, 1, or 3 mg/kg) immediately prior to the locomotor activity testing. SCH 23390 (0.06 mg/kg) was administered s.c. 30 min before α-PVP (3 mg/kg), PV8 (10 mg/kg), PV9 (10 mg/kg), methamphetamine (3 mg/kg) or saline injection. In the experiments with SCH 23390, the control mice received two injections of saline, 30 min apart. In all studies, horizontal activity and rearing (interruption of photocell beams in the bottom and top layers, respectively) were measured for 2 h within 10 min periods.

### Brain microdialysis

#### Surgery and microdialysis procedure

Animals (six per group) were anaesthetized with ketamine (7.5 mg/kg) and xylazine (1 mg/kg) and vertical microdialysis probes (MAB 10.8.2. Cu; AgnTho’s, Lidingö, Sweden) were implanted into the striatum using the following coordinates: AP + 1.0, L + 1.8, V − 3.8 [22]. On the next day, probe inlets were connected to a syringe pump (BAS, West Lafayette, IN, USA) which delivered an artificial cerebrospinal fluid composed of (mM): NaCl 147, KCl 2.7, MgCl_2_ 1.0, CaCl_2_ 1.2; pH 7.4, at a flow rate of 1.5 µL/min. After 1 h of washout, three basal dialysate samples were collected every 20 min. The animals were then injected s.c. with the appropriate drug, as indicated in the figure captions, and fractions collections continued for 180 min. At the end of the experiment, the mice were sacrificed, their brains were isolated and histologically examined to validate the probe placement.

#### Analytical procedure of samples

DA and 5-HT contents in the dialysate fractions were analyzed by HPLC with electrochemical detection. Chromatography was performed using an Ultimate 3000 System (Dionex, Sunnyvale, CA, USA), a Coulochem III electrochemical detector (model 5300; ESA, Chelmsford, MA, USA) with a 5020 guard cell, a 5014B microdialysis cell and a Hypersil Gold-C18 analytical column (3 × 100 mm; Thermo Scientific, Waltham, MA, USA). The mobile phase was composed of 0.1 M potassium phosphate buffer adjusted to pH 3.6, 0.5 mM EDTA, 16 mg/L 1-octanesulfonic acid sodium salt, and 2% methanol. The flow rate during analysis was set at 0.7 mL/min. The applied potential of a guard cell was + 600 mV, while those of microdialysis cells were: *E*_1_ = − 50 mV and *E*_2_ = + 300 mV with a sensitivity set at 50 nA/V. The chromatographic data was processed by Chromeleon v. 6.80 (Dionex) software, run on a personal computer.

### Data analysis

#### Locomotor activity

All statistical analyses were performed using GraphPad Prism 6.0 software (GraphPad, San Diego, CA, USA). Locomotor activity was expressed as the total distance travelled (cm) and total number of rearings during each 10-min bin during a 120-min session. A two-way repeated measure analysis of variance (treatment condition; time after injection) followed by Dunnett’s or Tukey’s post hoc test was conducted for horizontal and vertical activity in 10-min bins. Additionally, one-way ANOVA followed by Dunnett’s or Tukey’s post hoc test was performed for a total distance (cm) and total count of vertical beam breaks during a 120-min session.

#### Microdialysis

Statistical analysis was performed using STATISTICA V12.0 software (StatSoft, Kraków, Poland). Monoamines’ levels are expressed as percent of the basal level assumed as 100%. The statistical significance was calculated using a repeated measure ANOVA over 20-min bins for the time course, followed by Tukey’s post hoc test. To analyze differences in AUC, one-way ANOVA was performed, followed by Tukey’s post hoc test.

## Results

### Spontaneous locomotor activities

All tested compounds, α-PVP, PV8, PV9, and methamphetamine, produced dose- and time-dependent stimulation of horizontal locomotor activities. In contrast, only the pyrovalerones, i.e., not methamphetamine, produced a dose-dependent increase of vertical activities.

**Fig. 2:**
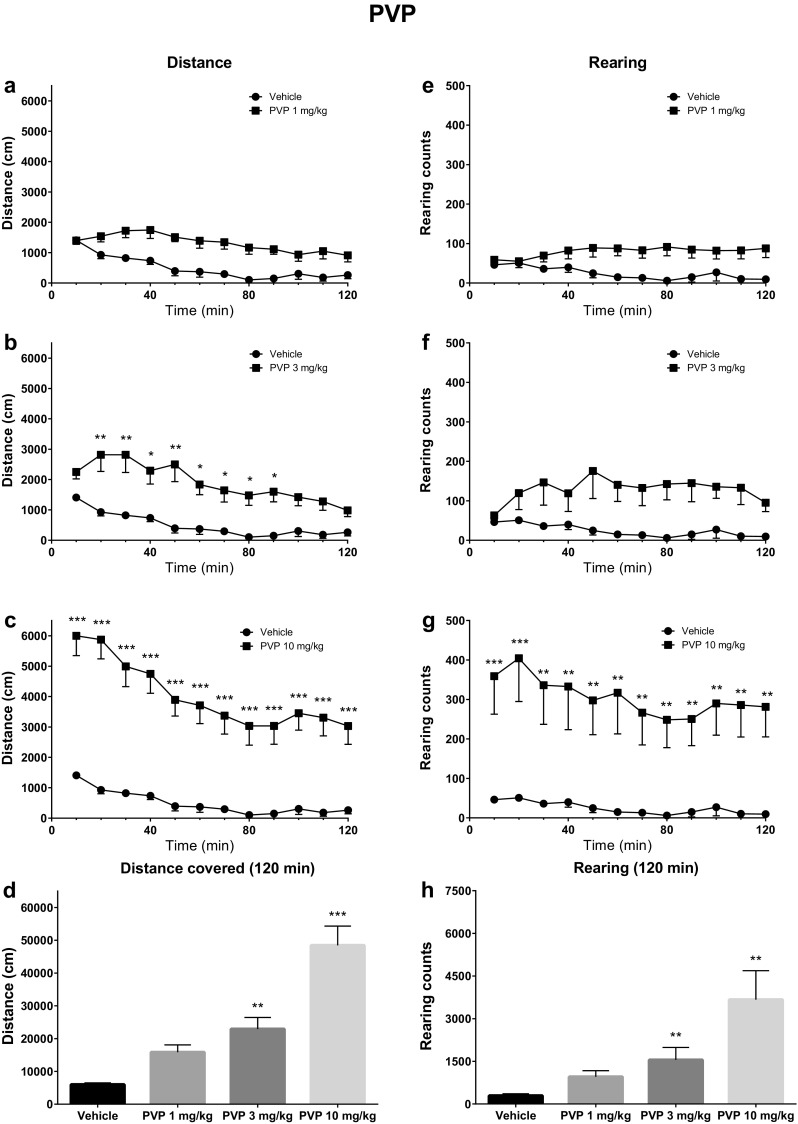
Spontaneous locomotor activities after treatment with α-PVP (PVP; 1, 3, 10 mg/kg). Average horizontal (**a**–**c**) and vertical (**e**–**g**) activities in 10-min bins. **d** Total distance travelled during 120 min. **h** Total rearing counts during 120 min. Data presented as mean ± standard error of the mean (SEM) (*n* = 8). ***p < 0.001; **p < 0.01; *p < 0.05 vs. control

The analysis of horizontal activities indicated significant effects of dose [*F*(3, 28) = 25.17; *p* < 0.0001], time [*F*(11, 308) = 19.57; *p* < 0.0001], and interaction between the factors [*F*(33, 308) = 2.98; *p* < 0.0001] for the α-PVP treatment. Post hoc analysis confirmed a statistically significant increase in locomotor activity vs. control group for 3 mg/kg α-PVP (started 10 min following injection and lasting for 80 min) and 10 mg/kg α-PVP (occurred within the first 10 min and lasting for 120 min) (Fig. 2). Additional analysis indicated that the total distance travelled in 120 min was significantly greater than for the vehicle group for both 3 mg/kg (*p* < 0.01) and 10 mg/kg (*p* < 0.001). Vertical activities were also significantly altered by α-PVP treatment, with a significant effect being observed for dose [*F*(3, 28) = 6.68; *p* = 0.0015], interaction [*F*(33, 308) = 2.106; *p* = 0.0006], but not time [*F*(11, 308) = 1.39; *p* > 0.05]. Post hoc analysis confirmed a significant difference in the number of rearings from the control group for α-PVP (10 mg/kg), which occurred immediately after injection and lasted for the full period of observation (120 min). An additional analysis of the total number of rearings within 120 min revealed a significant increase as compared to the control group after treatment with α-PVP at doses of 3 mg/kg (*p* < 0.01) and 10 mg/kg (*p* < 0.01) (Fig. 2).

**Fig. 3:**
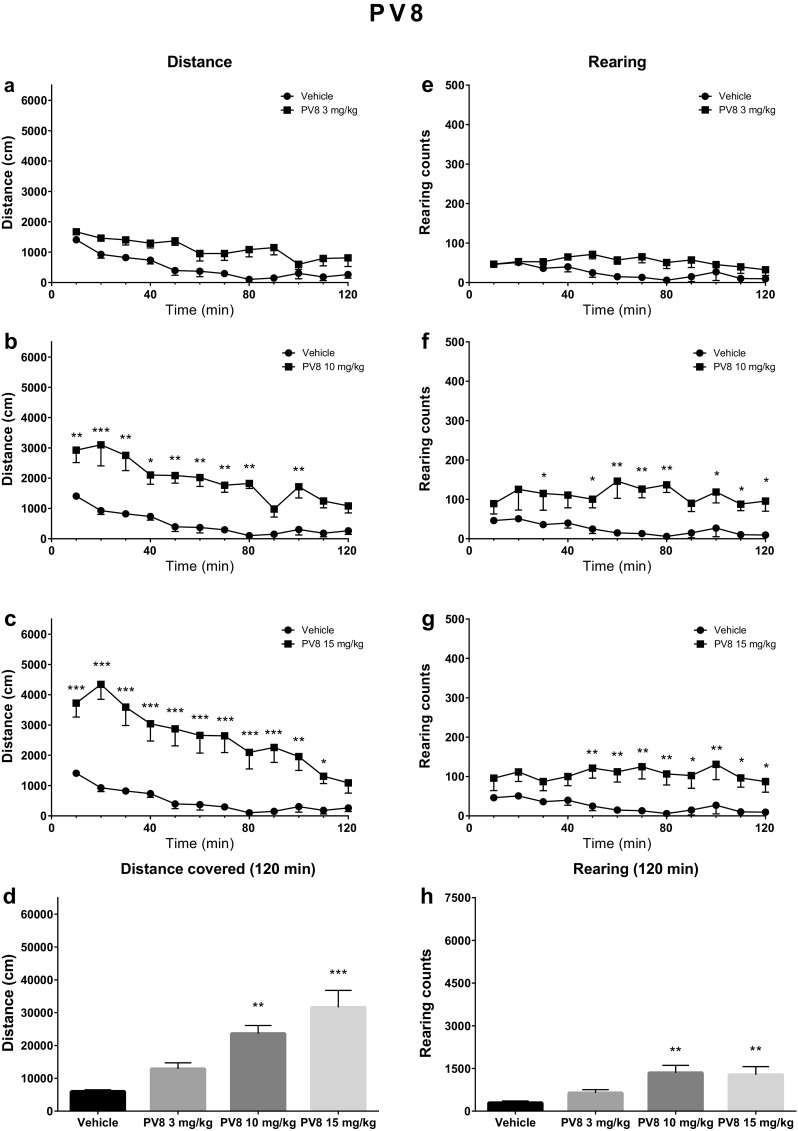
Spontaneous locomotor activities after treatment with PV8 (3, 10, 15 mg/kg). Average horizontal (**a**–**c**) and vertical (**e**–**g**) activities in 10-min bins. **d** Total distance travelled during 120 min. **h** Total rearing counts during 120 min. Data presented as mean ± SEM (*n* = 7–8). ***p < 0.001; **p < 0.01; *p < 0.05 vs. control

Initial analysis demonstrated that following the treatment with PV8, significant factors affecting horizontal activity were dose [*F*(3, 26) = 13.61; *p* < 0.0001], time [*F*(11, 286) = 24.60; *p* < 0.0001], and the interaction between these factors [*F*(33, 286) = 2.76; *p* < 0.0001]. Post hoc analysis revealed a significant increase of locomotor activities vs. control group following administration of 10 mg/kg (0–80 and 90–100 min after injection) and 15 mg/kg (0–110 min after injection) (Fig. 3). Analysis of the total distance covered in 120 min indicated a significant difference between the control group and the animals treated with PV8 at doses of 10 mg/kg (*p* < 0.01) and 15 mg/kg (*p* < 0.001). The vertical activities of mice were also affected by PV8 treatment; however, the only significant factor was dose [*F*(3, 26) = 6.09; *p* = 0.0028], but not time [*F*(11, 286) = 1.60; *p* > 0.05] nor interaction [*F*(33, 286) = 0.86; *p* > 0.05]. Post hoc analysis revealed the significantly increased numbers of rearings as compared to the control group after treatment with PV8 at 10 mg/kg (20–30, 40–80 and 90–120 min following administration) and 15 mg/kg (40–120 min following administration). Analysis of the total number of rearings within the 120 min period indicated a significant increase as compared to the control group after treatment with PV8 at doses of 10 mg/kg (*p* < 0.01) or 15 mg/kg (*p* < 0.01) (Fig. 3).

**Fig. 4:**
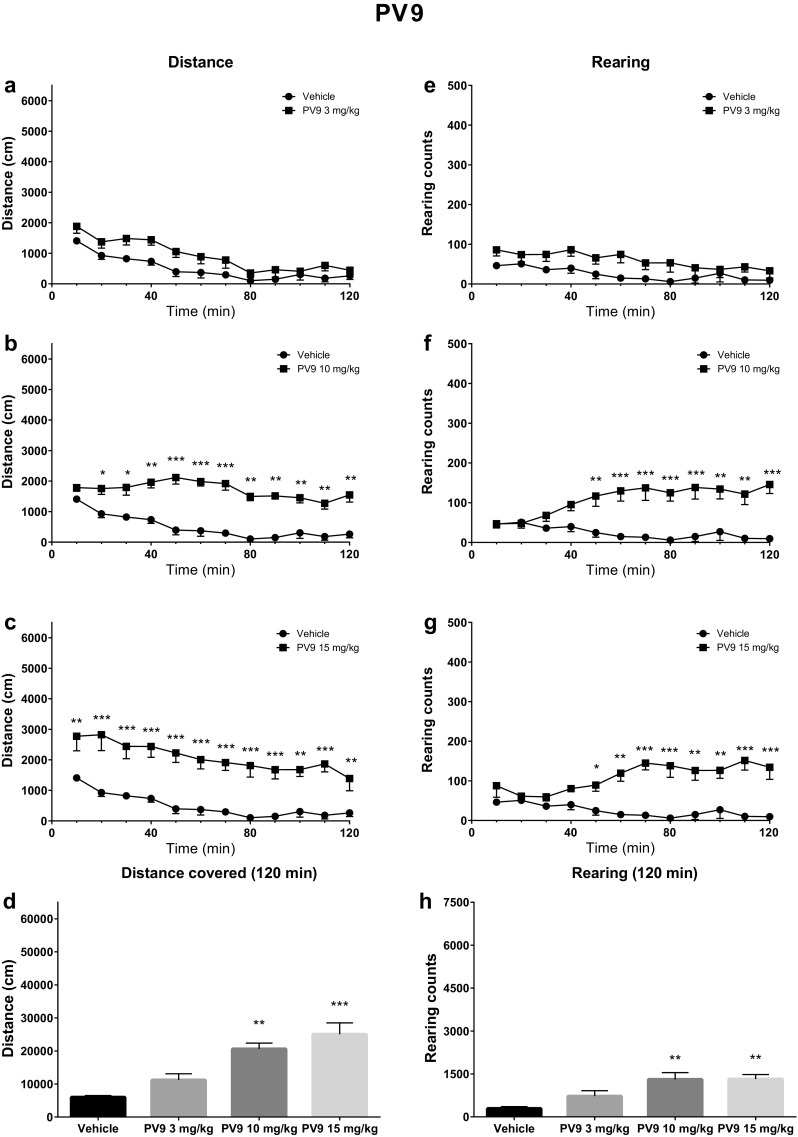
Spontaneous locomotor activities after treatment with PV9 (3, 10, 15 mg/kg). Average horizontal (**a**–**c**) and vertical (**e**–**g**) activities in 10-min bins. **d** Total distance travelled during 120 min. **h** Total rearing counts during 120 min. Data presented as mean ± SEM (*n* = 8). ***p < 0.001; **p < 0.01; *p < 0.05 vs. control

After PV9 injection, analysis indicated that dose [*F*(3, 28) = 16.02; *p* < 0.0001], time [*F*(11, 308) = 21.90; *p* < 0.0001], and interaction [*F*(33, 308) = 1.98; *p* = 0.0015] were significant factors. Post hoc analysis revealed a significant increase in the distance covered by the group receiving PV9 at 10 mg/kg (starting from 10 min after administration and lasting to the end of the experiment) and 15 mg/kg (through 0–120 min following administration) as compared to controls (Fig. 4). Additionally, the total distance covered during 120 min by the mice receiving PV9 at 10 mg/kg (*p* < 0.01) and 15 mg/kg (*p* < 0.001) was significantly greater than controls. Vertical activities were also altered by the administration of PV9, with dose [*F*(3, 28) = 7.95; *p* = 0.0005], time [*F*(11, 308) = 2.30; *p* = 0.0102], and interaction [*F*(33, 308) = 5.53; *p* < 0.0001] being significant factors. Post hoc analysis indicated a significant increase in the rearing counts after administration of PV9 at 10 mg/kg (40–120 min) and 15 mg/kg (40–120 min). Additionally, PV9 at 10 mg/kg (*p* < 0.01) and 15 mg/kg (*p* < 0.01) significantly increased the total rearing counts over 120 min vs. control group (Fig. 4).

**Fig. 5:**
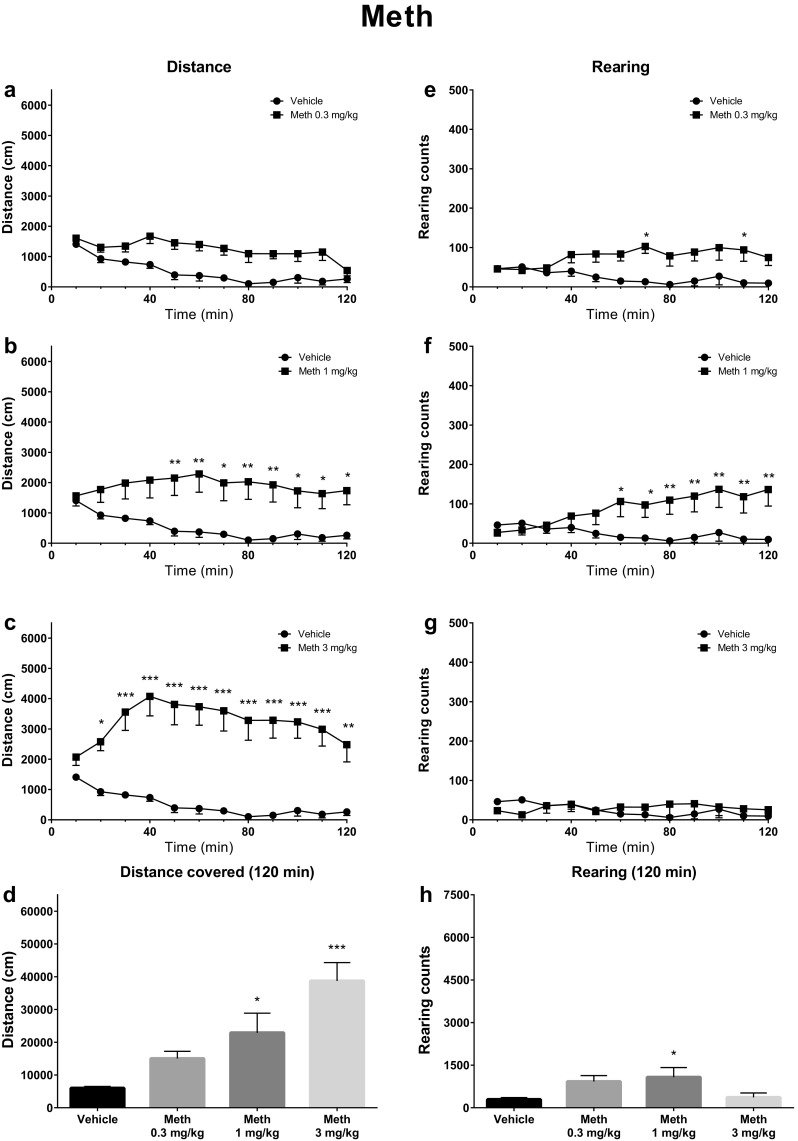
Spontaneous locomotor activities after treatment with methamphetamine (Meth; 0.3, 1, 3 mg/kg). Average horizontal (**a**–**c**) and vertical (**e**–**g**) activities in 10-min bins. **d** Total distance travelled during 120 min. **h** Total rearing counts during 120 min. Data presented as mean ± SEM (*n* = 8). ***p < 0.001; **p < 0.01; *p < 0.05 vs. control

Treatment of mice with methamphetamine resulted in increased horizontal locomotor activities vs. control group with dose [*F*(3, 28) = 10.66; *p* < 0.0001], time [*F*(11, 308) = 4.99; *p* < 0.0001], and interaction [*F*(33, 308) = 2.94; *p* < 0.0001] being significant factors. Post hoc analysis indicated elevated locomotor activity after 1 mg/kg (40–120 min) and 3 mg/kg (10–120 min) methamphetamine treatments. The total distance traveled in 120 min was significantly increased vs. control group by methamphetamine at 1 mg/kg (*p* < 0.05) and 3 mg/kg (*p* < 0.001). Methamphetamine also increased the vertical activities with dose [*F*(3, 28) = 3.31; *p* = 0.0345], time [*F*(11, 308) = 3.20; *p* < 0.0004], and interaction [*F*(33, 308) = 2.96; *p* < 0.0001] being significant factors. The number of rearings was significantly increased vs. control group after methamphetamine treatment at 0.3 mg/kg (60–70 and 100–110 min) and 1 mg/kg (50–120 min), but not at 3 mg/kg. During the 120-min experiment, the total number of rearings was observed to increase only for the 1 mg/kg dose (*p* < 0.05) (Fig. 5).

Comparison of effects produced by the three compounds (α-PVP, PV8 and PV9), used in the same dose of 10 mg/kg, on the horizontal activities showed significant effects of treatments [*F*(2, 20) = 15.55; *p* < 0.0001], time [*F*(11, 220) = 13.78; *p* < 0.0001], and interaction between factors [*F*(22, 220) = 3.42; *p* < 0.0001]. Post hoc analysis revealed significant differences between α-PVP vs. PV8 (0–70 and 80–120 min after injection) and α-PVP vs. PV9 (0–120 min after injection), but no difference was found between PV8 and PV9 (Fig. S1). The total distance traveled during 120 min was significantly lower in PV8 than in α-PVP (*p* < 0.01) and significantly lower in PV9 than in α-PVP (*p* < 0.01), but no difference was found between PV8 and PV9 (*p* > 0.05). The vertical activity analysis showed that treatment [*F*(2, 20) = 4.47; *p* = 0.0248] and interaction [*F*(22, 220) = 3.33; *p* < 0.0001] had significant effects, but not time [*F*(11, 220) = 0.69; *p* > 0.05]. Post hoc analysis indicated significant differences between α-PVP vs. PV8 (0–50 and 100–110 min after injection) and α-PVP vs. PV9 (0–40 min after injection). No difference between PV8 and PV9 was found. Analysis of total rearing counts during 120 min indicated a significant difference only between α-PVP and PV9 (*p* < 0.05) (Fig. S1).

The analysis of the distance traveled by mice following administration of each of the tested pyrovalerones vs. methamphetamine (all compounds were used at the same dose of 3 mg/kg) indicated that treatment [*F*(3, 27) = 11.92; *p* < 0.0001], time [*F*(11, 297) = 8.88; *p* < 0.0001], and interaction [*F*(33, 297) = 3.0; *p* < 0.0001] were significant factors. Post hoc analysis revealed that α-PVP (30–120 min after injection), PV8 (20–120 min after injection), and PV9 (20–120 min after injection) produced significantly lower locomotions than methamphetamine (Fig. S2). The total distance covered by mice during 120 min was also significantly lower after α-PVP (*p* < 0.05), PV8 (*p* < 0.01), and PV9 (*p* < 0.001) injections as compared to methamphetamine. Analysis of the vertical activities revealed that only treatment was a significant factor [*F*(3, 27) = 3.78; *p* = 0.0219], but not time [*F*(11, 297) = 1.75; *p* > 0.05] or interaction [*F*(33, 297) = 1.20; *p* > 0.05]. Only α-PVP treatment was associated with a significantly higher number of rearings as compared to methamphetamine (10–30 and 40–110 min after injection). The total number of rearings during 120 min was also significantly higher vs. methamphetamine only after α-PVP treatment (*p* < 0.01) (Fig. S2).

**Fig. 6:**
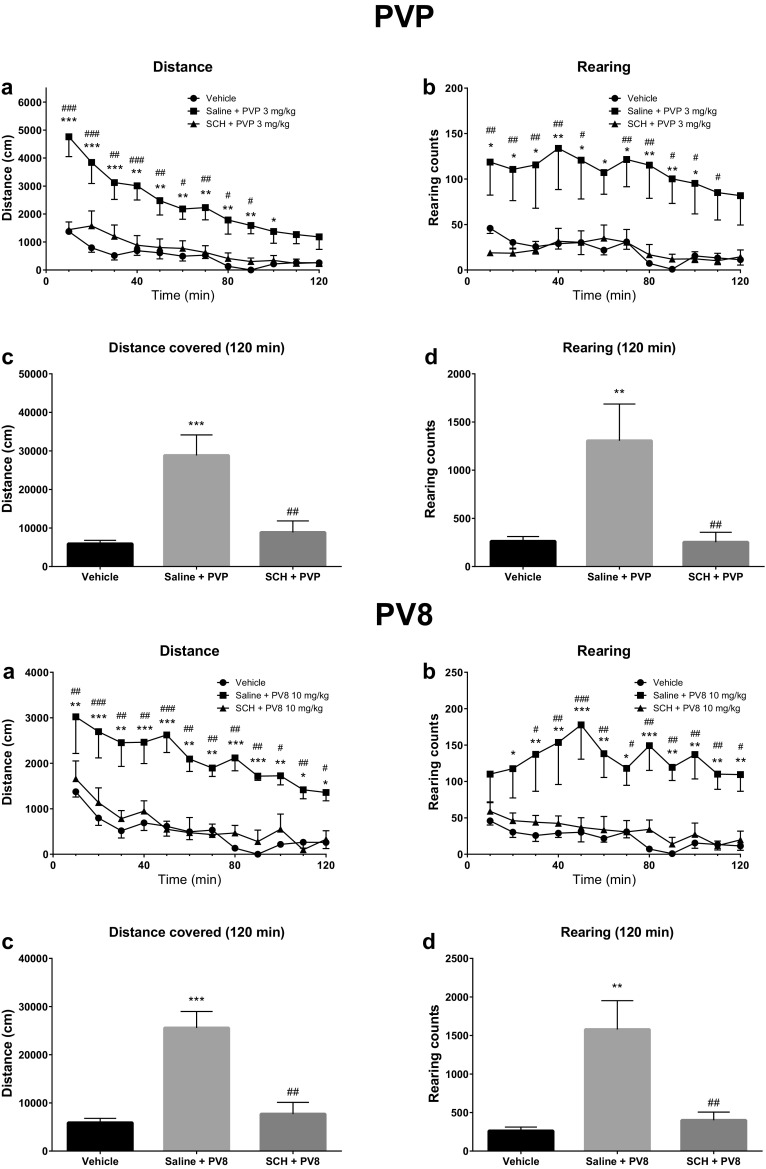
Spontaneous locomotor activities after treatment with α-PVP (PVP; 3 mg/kg) (upper half) or PV8 (10 mg/kg) (lower half), preceded by SCH 23390 (0.06 mg/kg) pretreatment. Average horizontal (**a**) and vertical (**b**) activities in 10-min bins. Total distance travelled (**c**) and total rearing counts (**d**) during 120 min. Data presented as mean ± SEM (*n* = 8). ***p < 0.001; **p < 0.01; *p < 0.05 against vehicle control, ^###^*p* < 0.001; ^##^*p* < 0.01; ^#^*p* < 0.05 against SCH 23390 pretreated group

**Fig. 7:**
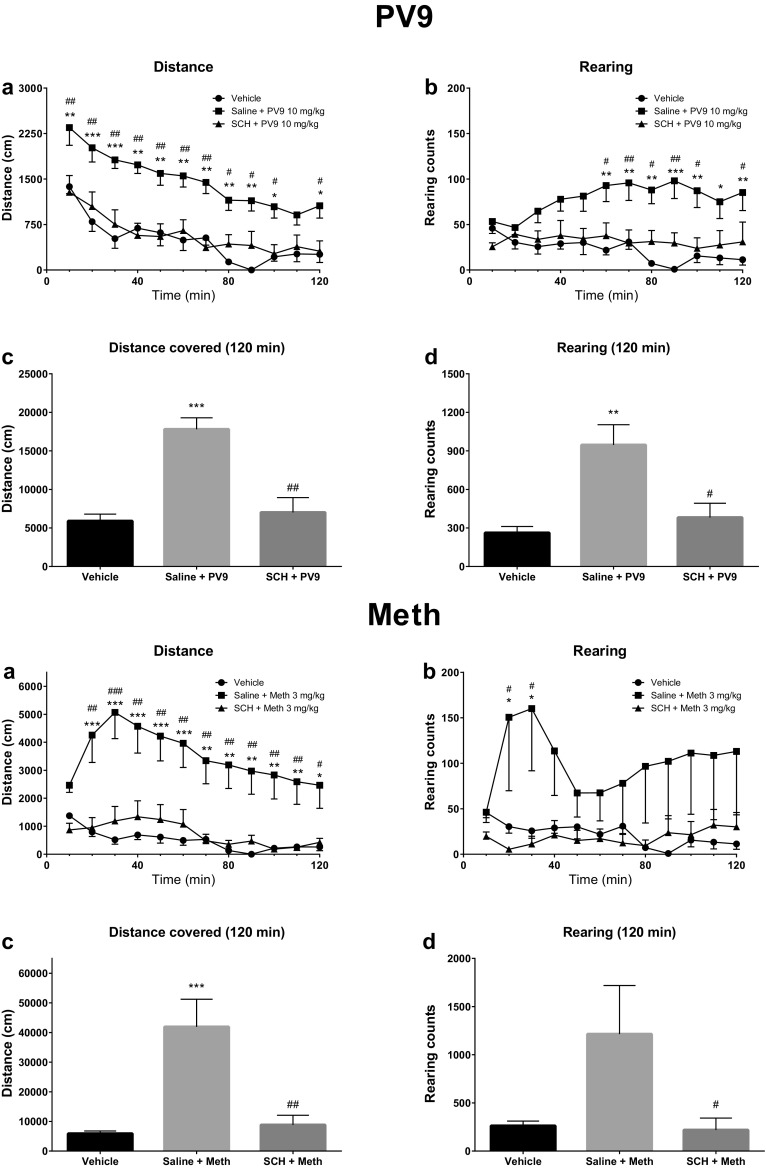
Spontaneous locomotor activities after treatment with PV9 (10 mg/kg) (upper half) or methamphetamine (Meth; 3 mg/kg) (lower half), preceded by SCH 23390 (0.06 mg/kg) pretreatment. Average horizontal (**a**) and vertical (**b**) activities in 10-min bins. Total distance travelled (**c**) and total rearing counts (**d**) during 120 min. Data presented as mean ± SEM (*n* = 8–16). ***p < 0.001; **p < 0.01; *p < 0.05 against vehicle control, ^###^*p* < 0.001; ^##^*p* < 0.01; ^#^*p* < 0.05 against SCH 23390 pretreated group

Pretreatment of mice with a selective D_1_-DA receptor antagonist SCH 23390 (0.06 mg/kg) potently reduced the stimulatory effects of α-PVP (3 mg/kg), PV8 (10 mg/kg), PV9 (10 mg/kg), and methamphetamine (3 mg/kg) on the horizontal locomotor activity. Moreover, the stimulation of vertical activity caused by α-PVP, PV8 and PV9 was blocked by SCH 23390 (Figs. 6, 7).

Analysis of groups receiving either SCH 23390 + α-PVP or saline + α-PVP indicated that treatment [*F*(2, 21) = 12.28; *p* = 0.0003], time [*F*(11, 231) = 33.96; *p* < 0.0001], and interaction [*F*(22, 231) = 5.35; *p* < 0.0001] were significant factors determining horizontal activities. Post hoc analysis showed that locomotor activity of mice was significantly lower in SCH 23390 + α-PVP as compared to the saline + α-PVP group in 0–90 min after α-PVP injection (Fig. 6, upper half). Additionally, the total distance covered during 120 min was also significantly lowered by SCH 23390 pretreatment as compared to the saline + α-PVP group (*p* < 0.01). The vertical activities of mice were also significantly determined by treatment [*F*(2, 21) = 6.95; *p* = 0.0048], time [*F*(11, 231) = 2.87; *p* = 0.0015], but not interaction between factors [*F*(22, 231) = 0.56; *p* > 0.05]. Post hoc analysis revealed that the rearing counts were significantly lower in the SCH 23390 + α-PVP group as compared to the saline + α-PVP group 0–50 and 60–110 min after α-PVP injection. Similarly, the total number of rearings during 120 min was significantly lowered by SCH 23390 pretreatment (*p* < 0.01) (Fig. 6, upper half).

The analysis of groups receiving either SCH 23390 + PV8 or saline + PV8 indicated that treatment [*F*(2, 21) = 19.42; *p* < 0.0001], time [*F*(11, 231) = 12.34; *p* < 0.0001], but not interaction [*F*(22, 231) = 0.79; *p* > 0.05] were significant factors determining horizontal locomotor activities. Post hoc analysis revealed that distance traveled by mice was significantly reduced by SCH 23390 in every 10-min bin during the whole observation time (Fig. 6, lower half). Additionally, the total distance covered during 120 min was also reduced by SCH 23390 pretreatment (*p* < 0.01). SCH 23390 also reduced the PV8-induced increase of vertical activities, depending on treatment [*F*(2, 21) = 10.22; *p* = 0.0008] and time [*F*(11, 231) = 2.22; *p* = 0.0142], but not interaction between factors [*F*(22, 231) = 1.01; *p* > 0.05]. The number of rearings was constantly reduced in the SCH 23390-pretreated group vs. the saline + PV8 group, starting at 20 min after PV8 injection. Moreover, SCH 23390 significantly reduced the total number of rearings through 120 min as compared to the saline + PV8 group (*p* < 0.01) (Fig. 6, lower half).

Pretreatment with SCH 23390 significantly reduced elevation in mice horizontal activities produced by PV9, depending on treatment [*F*(2, 29) = 19.28; *p* < 0.0001] and time [*F*(11, 319) = 14.42; *p* < 0.0001], but not interaction between factors [*F*(22, 319) = 0.68; *p* > 0.05]. The distance covered in 10-min bins was significantly lower in the SCH 23390 + PV9 than in the saline + PV9 group (0–100 and 110–120 min after PV9 injection) (Fig. 7, upper half). Also the total distance covered during 120 min was significantly lower in SCH 23390 + PV9 as compared to the saline + PV9 group (*p* < 0.01). The vertical activities of mice were significantly determined by treatment [*F*(2, 29) = 6.73; *p* = 0.004] and interaction between factors [*F*(22, 319) = 2.08; *p* = 0.0034], but not time [*F*(11, 319) = 0.58; *p* > 0.05]. The number of rearings was lower in the SCH 23390 + PV9 than in the saline + PV9 group during two periods of the experiment: 50–100 and 110–120 min after PV9 injection, and the total number of rearings during 120 min was also significantly reduced in the SCH 23390 + PV9 as compared to the saline + PV9 group (*p* < 0.05) (Fig. 7, upper half).

Pretreatment with SCH 23390 significantly reduced the elevation in horizontal activities produced by methamphetamine, depending on treatment [*F*(2, 20) = 11.83; *p* = 0.0004], time [*F*(11, 220) = 10.44; *p* < 0.0001], and interaction between factors [*F*(22, 220) = 3.55; *p* < 0.0001]. Post hoc analysis indicated that the distance covered was significantly lower in the SCH 23390 + methamphetamine group as compared to saline + methamphetamine, starting at 10 min after methamphetamine injection, and lasting to the end of the experiment (Fig. 7, lower half). Also the total distance traveled during 120 min was significantly lower in SCH 23390 + methamphetamine as compared to the saline + methamphetamine group (*p* < 0.01). After treatment of mice with either saline + methamphetamine or SCH 23390 + methamphetamine, neither treatment [*F*(2, 20) = 3.29; *p* > 0.05], time [*F*(11, 220) = 0.48; *p* > 0.05], nor interaction [*F*(22, 220) = 0.80; *p* > 0.05] played a significant role in their vertical activities. Post hoc analysis revealed significant differences (*p* < 0.05) between saline + methamphetamine and saline + saline, and between saline + methamphetamine and SCH 23390 + methamphetamine, at only 10–20 and 20–30 min following administration of methamphetamine. Neither methamphetamine-treated groups significantly differed as compared to saline in the total number of rearings during 120 min; however, there was a significant difference (*p* < 0.05) between saline + methamphetamine and SCH 23390 + methamphetamine (Fig. 7, lower half).

### Extracellular levels of DA and 5-HT in the mouse striatum

Across all microdialysis experiments, baseline extracellular levels (in pg/10 μL; mean ± standard error of mean) were 6.98 ± 0.56 (*n* = 36) for DA and 0.37 ± 0.02 (*n* = 36) for 5-HT. Figure S3 shows microdialysis probe placements.

**Fig. 8:**
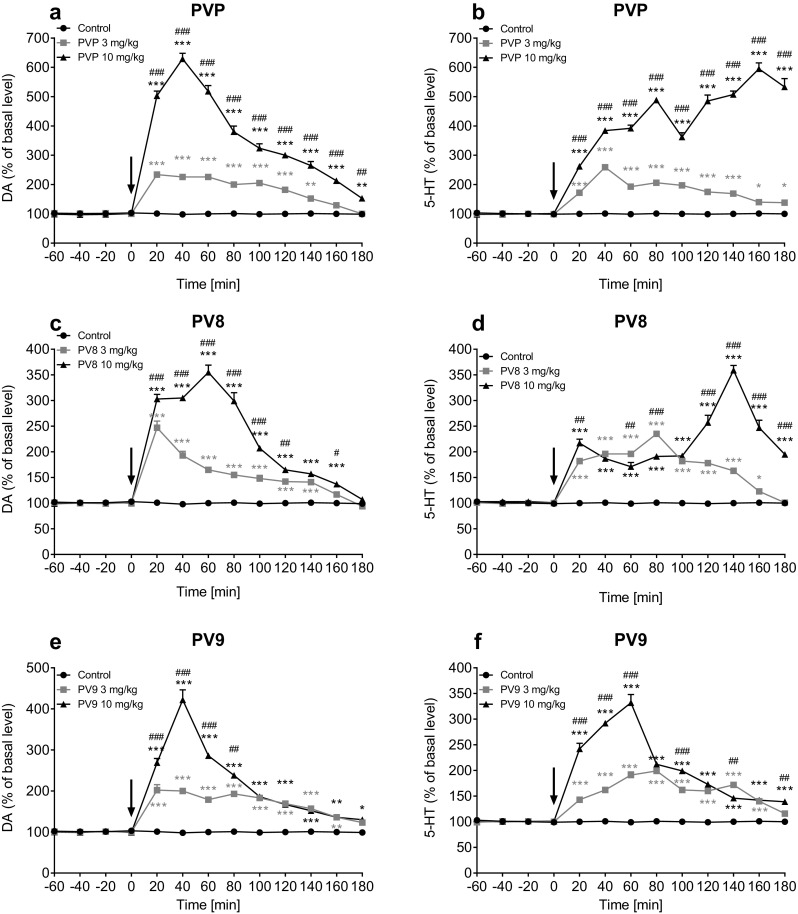
Effects of α-PVP (PVP), PV8 and PV9 on the extracellular level of dopamine (DA) (**a**, **c**, **e**) and serotonin (5-HT) (**b**, **d**, **f**) in mouse striatum shown as a time-course. Drug administration is indicated with an arrow. Data expressed as mean ± SEM (*n* = 6). ***p < 0.001; **p < 0.01; *p < 0.05 against control group, ^###^*p* < 0.001; ^##^*p* < 0.01; ^#^*p* < 0.05 between 3 and 10 mg/kg groups

The administration of α-PVP at doses of 3 and 10 mg/kg increased extracellular DA levels in the mouse striatum, with the maximum effects at ca. 230 and ca. 630% of the basal level after 20 and 40 min, respectively (Fig. 8a). Repeated measure ANOVA indicated that treatment [*F*(2, 15) = 459; *p* < 0.0001], time [*F*(8, 120) = 257; *p* < 0.0001], and interaction between factors [*F*(16, 120) = 130; *p* < 0.0001] had significant effects. Treatment with α-PVP also significantly elevated 5-HT concentrations, with the maximum at ca. 260% of the basal level after 40 min at 3 mg/kg, and with maximum at ca. 600% of the basal level 160 min after treatment with 10 mg/kg (Fig. 8b). Statistical analysis indicated that treatment [*F*(2, 15) = 1553; *p* < 0.0001], time [*F*(8, 120) = 31; *p* < 0.0001], and interaction [*F*(16, 120) = 52; *p* < 0.0001] were significant factors.

The treatment of mice with PV8 at both doses (3 and 10 mg/kg) caused an increase of extracellular DA level in the mouse striatum, with the maximum effects at ca. 250% of the basal level 20 min after exposure, and at ca. 360% of the basal level after 60 min, respectively (Fig. 8c). Repeated measure ANOVA revealed significant effects of treatment [*F*(2, 15) = 717; *p* < 0.0001], time [*F*(8, 120) = 206; *p* < 0.0001], and interaction between factors [*F*(16, 120) = 91; *p* < 0.0001]. PV8 also significantly increased 5-HT levels at 3 and 10 mg/kg, with the maximum effects at ca. 240% of the basal level after 80 min, and at ca. 360% of the basal level after 140 min, respectively (Fig. 8d). Repeated measure ANOVA indicated that treatment [*F*(2, 15) = 1577; *p* < 0.0001], time [*F*(8, 120) = 48; *p* < 0.0001], and interaction between factors [*F*(16, 120) = 71; *p* < 0.0001] were significant factors.

The treatment with PV9 evoked an increase of extracellular DA level at both examined doses (3 and 10 mg/kg), with the maximum at ca. 200% of the basal level after 20 min, and at ca. 430% of the basal level after 40 min, respectively (Fig. 8e). Repeated measure ANOVA revealed significant effects of treatment [*F*(2, 15) = 291; *p* < 0.0001], time [*F*(8, 120) = 97; *p* < 0.0001], and interaction between factors [*F*(16, 120) = 55; *p* < 0.0001]. A significant increase in 5-HT concentrations was observed after treatment with both 3 and 10 mg/kg PV9, with the maximal effects at ca. 200% of the basal effect after 80 min, and at ca. 330% of the basal level after 60 min after injection, respectively (Fig. 8f). Statistical analysis revealed the significance of treatment [*F*(2, 15) = 457; *p* < 0.0001], time [*F*(8, 120) = 107; *p* < 0.0001], and interaction between factors [*F*(16, 120) = 68; *p* < 0.0001].

**Fig. 9:**
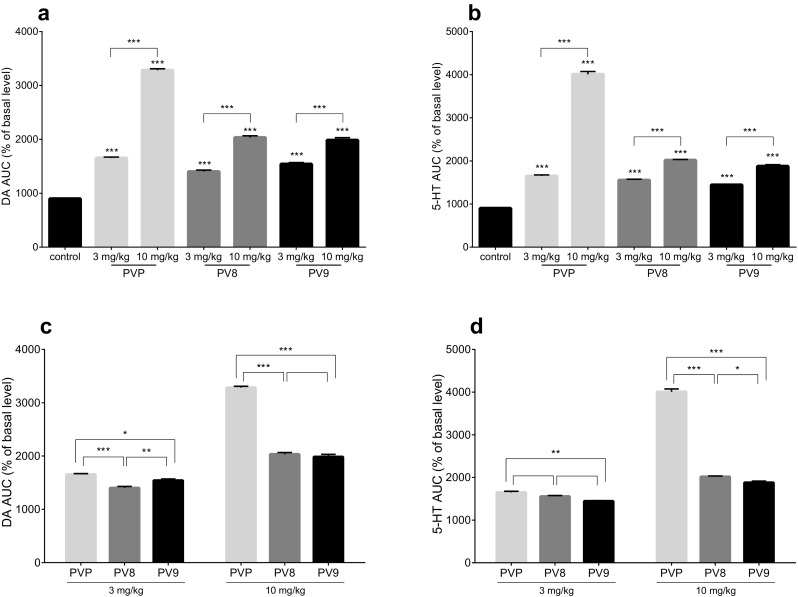
Total effects of α-PVP (PVP; 3 and 10 mg/kg), PV8 (3 and 10 mg/kg) and PV9 (3 and 10 mg/kg) on the extracellular levels of DA (**a**) and 5-HT (**b**) in mouse striatum. Comparison of potency to increase extracellular levels of DA (**c**) and 5-HT (**d**) among α-PVP (3 and 10 mg/kg), PV8 (3 and 10 mg/kg) and PV9 (3 and 10 mg/kg). Data expressed as the area under the curve (AUC) of DA and 5-HT levels during 180 min (mean ± SEM, *n* = 6). ***p < 0.001; **p < 0.01; *p < 0.05 against control group, unless otherwise specified

Additional one way ANOVA with Tukey post hoc analysis of the total effects measured as area under the curve (AUC) of both DA and 5-HT levels indicated that all the tested pyrovalerones, α-PVP, PV8 and PV9, used at doses of 3 and 10 mg/kg, caused a significant increase of DA (Fig. 9a) and 5-HT (Fig. 9b) concentrations over a 180-min period (*p* < 0.001). In all cases the effect produced by 10 mg/kg was significantly higher than that of 3 mg/kg (*p* < 0.001). Moreover, α-PVP at 3 and 10 mg/kg produced a significantly more pronounced increase in extracellular level of DA than PV8 (*p* < 0.001 and *p* < 0.001, respectively) and PV9 (*p* < 0.05 and *p* < 0.001, respectively) (Fig. 9c). The increase of 5-HT levels produced by α-PVP at 3 mg/kg was significantly higher than PV9 (*p* < 0.01), but not PV8 (*p* > 0.05); while at 10 mg/kg, α-PVP evoked a significantly stronger effect than both PV8 (*p* < 0.001) and PV9 (*p* < 0.001) (Fig. 9d).

## Discussion

All tested α-pyrrolidinophenones: α-PVP, PV8 and PV9, produced the dose-dependent increase of both horizontal and vertical spontaneous locomotor activities in mice, and increase of extracellular levels of DA and 5-HT in the striatum. When used at the tested highest dose, each compound produced psychomotor stimulation that lasted for at least 110 min, while elevation of monoamine levels persisted for at least 160 min. The psychostimulant effects of all the tested compounds increased with the dose, and no inverted U-shaped dose-effect curve was observed, which is characteristic of many stimulants applied in a wide dosing range [19, 23, 24]; however an exception was found for methamphetamine, which induced an increase in vertical activity at a dose of 1 mg/kg, but not at 3 mg/kg. Locomotor stimulation produced by α-PVP (10 mg/kg) was significantly more pronounced than that induced by PV8 (10 mg/kg) or PV9 (10 mg/kg), with no significant difference between PV8 and PV9 (both at 10 mg/kg). Moreover, α-PVP at the tested two doses (3 and 10 mg/kg) produced elevations of extracellular DA levels in the mouse striatum, which is considered to underlie a psychomotor stimulation [2], with a higher potency than PV8 and PV9.

These observations contrast with reports on the in vitro pharmacological evaluation of α-PVP and PV8 activities, where both drugs were found to have similar ability to inhibit DAT [12], and the suggestion that the increase of the side-chain bulk and lipophilicity should increase the potency of the drug [13]. Based on our in vivo findings and those of the mentioned in vitro studies [12, 13], we suggest that the psychostimulant potency of α-pyrrolidinophenones increase with the side chain length only for a limited range: α-PVP or α-PHP demonstrate the optimal range, and further extension of the side chain leads to a decline in psychomotor stimulant activity. However, our results on the potency of these drugs are in line with anecdotal data found on websites and forums devoted to NPS consumption. Users claim that PV8 and PV9 are much weaker drugs than α-PVP and 3,4-MDPV, and recommend higher doses to new users [14–17]. We must stress that anecdotal self-reports do not provide reliable data for many reasons. First of all, NPS users can never be sure of what compound they ingest, its chemical purity, and whether there are any adulterants in the product. Furthermore, phenomena such as multi-drug consumption and tolerance developing against substances with a similar mechanism of action may impede objective evaluation of the drug potency, especially even if the user has a long experience of psychostimulant abuse.

Possible explanations of the aforementioned variation in the potency could include the difference in pharmacokinetics of these substances, resulting in unequal concentrations being reached in the CNS over time. Absorption, distribution, hepatic metabolism and renal elimination are major processes affecting the concentration of a drug at the site of its action. The length of the aliphatic side chain affects the lipophilicity of the compound, which in turn may affect its absorption after administration, and favors distribution into lipophilic tissues, such as the CNS. Unfortunately, no study data could be found on the time-course of CNS concentration of α-pyrrolidinophenones after controlled administration. Most likely, the metabolism of pyrovalerone derivatives should not markedly affect their psychostimulant potency, as α-PVP-induced locomotor stimulation was found to be stronger than that evoked by PV8 and PV9 in almost every 10-min bin through the 120-min experiment, without any dramatic difference in the effect duration. Similarly, elevations in extracellular DA levels were observed quickly after treatment with 10 mg/kg of the tested pyrovalerones, with the maximal peak observed between 40 and 60 min after injection, and the level remained significantly elevated as compared to the basal level until 160–180 min of the experiment. However, the maximal DA level after treatment with α-PVP was almost twofold higher than after PV8, and a significant difference was observed in the AUC of DA concentrations.

The fact that the locomotor stimulation induced by pyrovalerones was completely abolished by the D_1_-DA receptor antagonist, SCH 23390 (0.06 mg/kg), to a level not higher than the baseline indicates that dopaminergic mechanisms are responsible. This observation is in line with results obtained by other research groups, where α-PVP-induced locomotor stimulation was also abolished by SCH 23390 [20, 21]. Kaizaki et al. [20] reported that SCH 23390 only partially abolished the effect of α-PVP to a level significantly higher than control, which may suggest that other mechanisms are also responsible for locomotor activation. However, the failure to fully inhibit the locomotor stimulation produced by a high α-PVP dose by a similar dose of SCH 23390 (60 μg/kg in this study vs. 50 μg/kg in a study by Kaizaki et al.) may be attributed to the fact that the dose of α-PVP differed by almost one order of magnitude between the studies: i.e., 3 mg/kg in the present study as compared to 25 mg/kg in experiments performed by Kaizaki et al. [20].

The stronger psychomotor stimulation and greater increase in DA levels associated with α-PVP as compared to PV8 or PV9 may be attributed to the difference between the potency of each of the tested α-pyrrolidinophenones to inhibit the human DAT used in in- vitro studies [12] and that to inhibit the mouse DAT in vivo used in the current study.

Interestingly, effects of all of the tested α-pyrrolidinophenones on horizontal locomotor activities were significantly lower than those produced by methamphetamine used at the same dose (3 mg/kg) (Fig. S2). This observation cannot be explained by the drugs’ affinity toward DAT or the potential to inhibit it; i.e., α-PVP was reported to inhibit DAT with IC_50_ not markedly higher than methamphetamine [12]. It rather illustrates the mutual mechanisms of action of methamphetamine, such that, in addition to being an inhibitor of monoamine reuptake, it is also a strong releaser of DA and NE [10–12].

Based on the in vitro pharmacologic profiles, pyrovalerones should not have significant affinity toward SERT or cause 5-HT efflux [12]. However, our study demonstrated that α-PVP, PV8 and PV9 produced a dose-dependent increase of extracellular 5-HT level in the mouse striatum (Figs. 8, 9). This observation could be explained by the presence of a functional DA-5-HT cross-talk, found both in human and rodent neurons, and described by Larsen et al. [25]. First, it is known that 5-HT can be accumulated in neurons by DAT, which itself is blocked by pyrovalerone derivatives. Second, SERT can also clear up DA, and at high concentrations, DA can competitively inhibit 5-HT uptake, albeit with a low potency. Additionally, it was demonstrated that elevated extracellular levels of DA can lead to the efflux of intracellular 5-HT via SERT during inward uptake of DA through this transporter [25]. In the present study after treatment of mice with 10 mg/kg of either α-PVP, PV8 or PV9, the maximal concentration of extracellular 5-HT in the striatum was observed always after the maximum DA peak (Fig. 8). Therefore, we suggest that the increase in extracellular 5-HT level is secondary to the elevation of DA. Our observations are in line with those of Matsumoto et al. [26], who found that methamphetamine, another DAT/NET-selective compound with no significant activity at SERT [12], could also elevate in vivo the extracellular level of 5-HT in the rat brain [26].

## Conclusions

Each year new members of synthetic cathinones appear on the NPS market. Of these, α-pyrrolidinophenones form a group endowed with strong psychostimulant effects. The present study describes for the first time in vivo assessment of the psychomotor stimulatory effects of two novel compounds from this group, PV8 and PV9. Contrary to predictions based on in vitro binding and uptake assays, we found that the longer side-chain compounds, such as PV8 and PV9, induce markedly weaker stimulation of mice locomotion and less pronounced elevation of extracellular DA levels in the mouse striatum, as compared to α-PVP. Additionally, the pyrovalerone analogs significantly elevated extracellular 5-HT levels. This study confirms that the enhancement of dopaminergic neurotransmission plays a dominant role in the psychomotor stimulation caused by α-PVP, PV8 and PV9, as the selective D_1_-DA receptor antagonist abolishes the stimulatory effect of the tested pyrovalerones. Moreover, when used at the same dose, all pyrovalerone derivatives produced effects significantly weaker than methamphetamine, an old-generation psychostimulant. Taken together, our findings highlight the necessity of in vivo assessment, along with in vitro experiments, in order to fully evaluate the pharmacological properties of NPS.

## Electronic supplementary material

Below is the link to the electronic supplementary material.

Supplementary material 1 (PDF 52 kb)

Supplementary material 2 (PDF 54 kb)

Supplementary material 3 (DOC 728 kb)
