# Supplementary material for: Effects of the new generation α-pyrrolidinophenones on spontaneous locomotor activities in mice, and on extracellular dopamine and serotonin levels in the mouse striatum
Source: Forensic Toxicol. 2018 Feb 26;36(2):334–50. doi: 10.1007/s11419-018-0409-x (PMC6002449; doi:10.1007/s11419-018-0409-x)
Supplement: Supplementary file 1 — Supplementary material 1 (PDF 52 kb) [file 11419_2018_409_MOESM1_ESM.pdf]

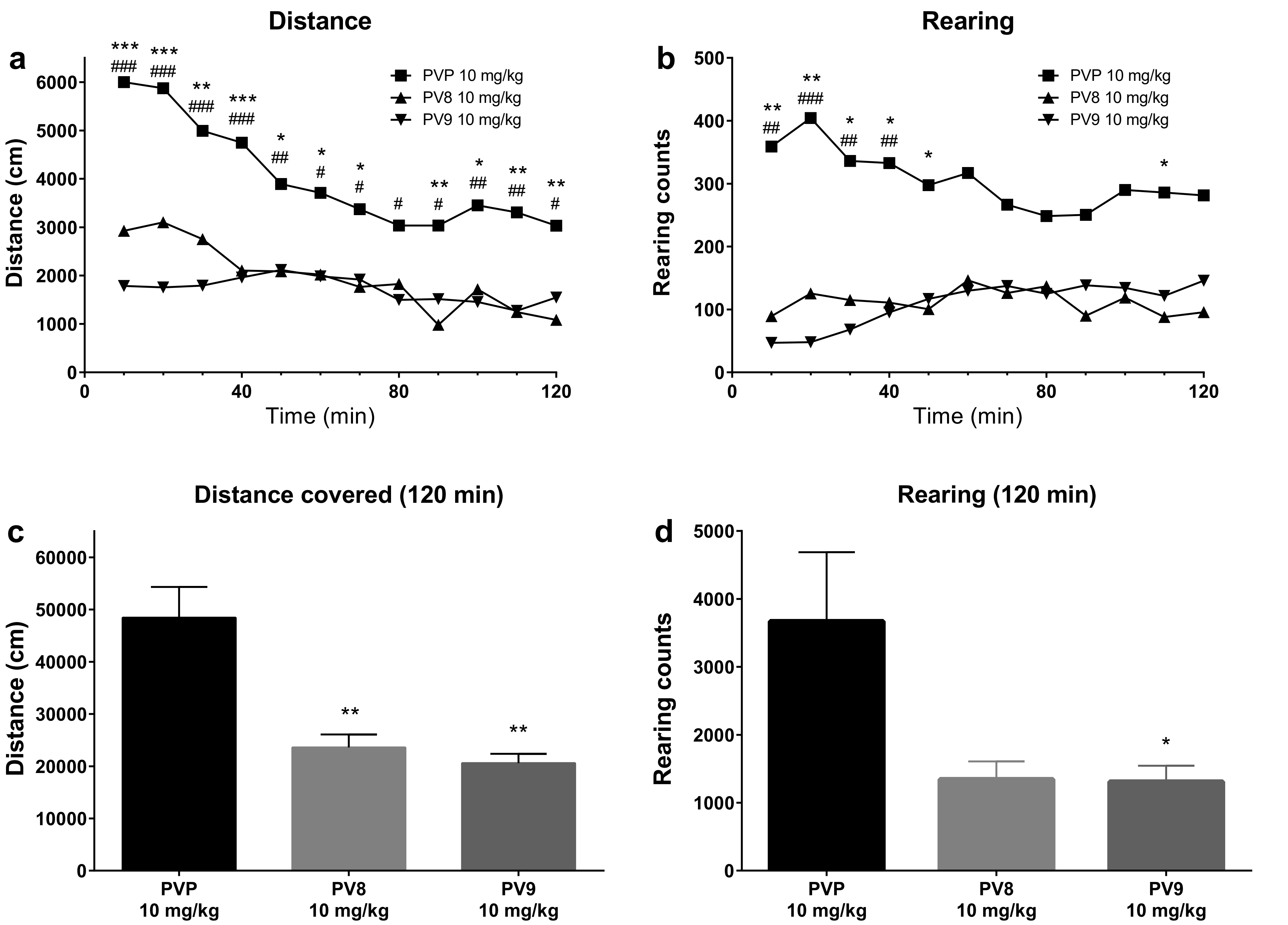

Fig. S1 Comparison of potency to increase spontaneous locomotor activities in mice of alpha-PVP (PVP, 10 mg/kg), PV8 (10 mg/kg) and PV9 (10 mg/kg). Average horizontal (**a**) and vertical (**b**) activities in 10-min bins. Data presented as mean (n = 7-8). *** $p < 0.001$; ** $p < 0.01$; * $p < 0.05$ against PV8, ### $p < 0.001$; ## $p < 0.01$; # $p < 0.05$ against PV9 during the same time bin. Total distance travelled (**c**) and total rearing counts (**d**) during 120 min. Data presented as mean +/- SEM (n = 7-8). ** $p < 0.01$; * $p < 0.05$ against alpha-PVP
