# Supplementary material for: Effects of the new generation α-pyrrolidinophenones on spontaneous locomotor activities in mice, and on extracellular dopamine and serotonin levels in the mouse striatum
Source: Forensic Toxicol. 2018 Feb 26;36(2):334–50. doi: 10.1007/s11419-018-0409-x (PMC6002449; doi:10.1007/s11419-018-0409-x)
Supplement: Supplementary file 2 — Supplementary material 2 (PDF 54 kb) [file 11419_2018_409_MOESM2_ESM.pdf]

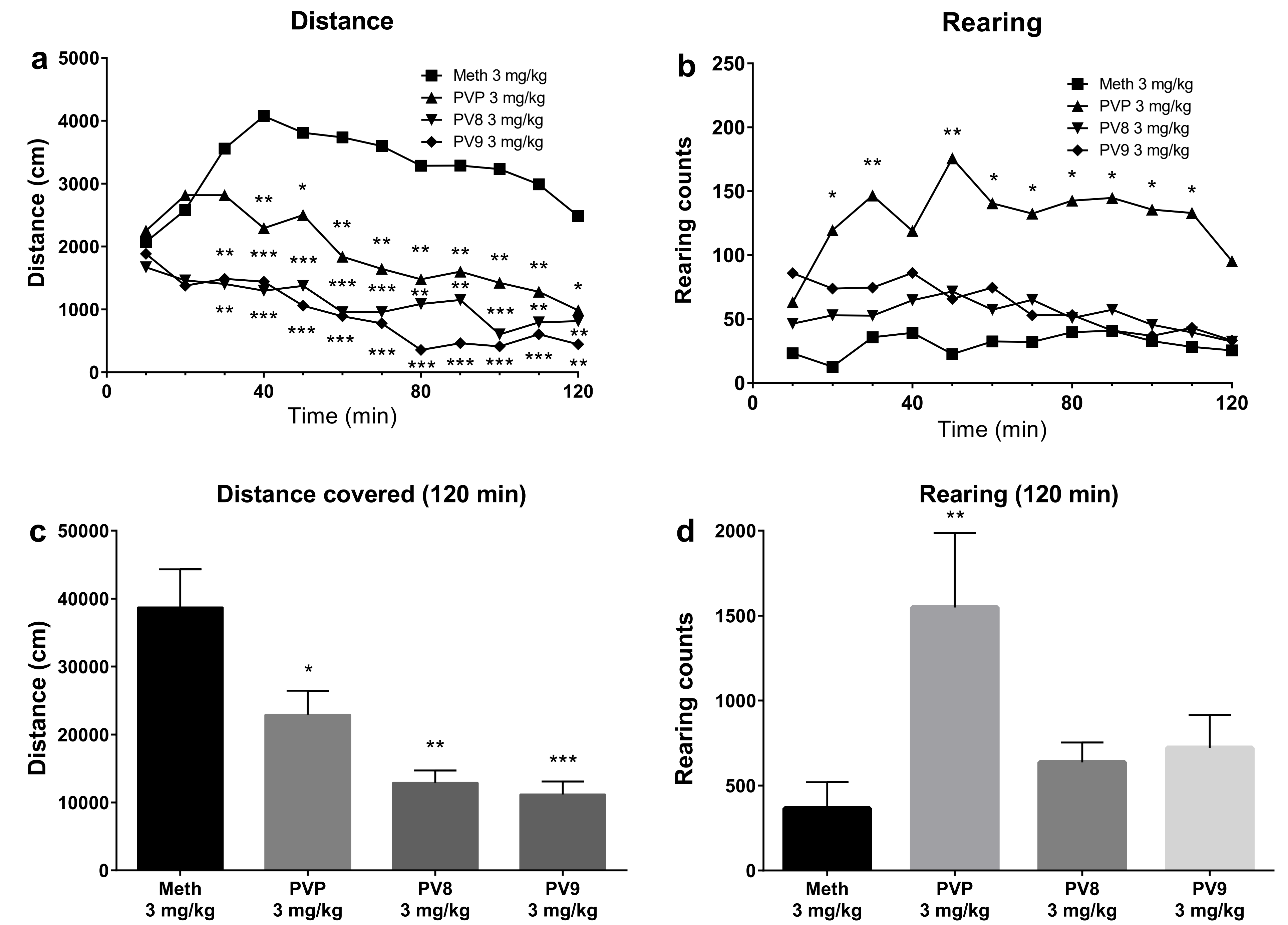

Fig. S2 Comparison of potency to increase spontaneous locomotor activities in mice of alpha-PVP (PVP, 3 mg/kg), PV8 (3 mg/kg) and PV9 (3 mg/kg) with methamphetamine (meth, 3 mg/kg). Average horizontal (**a**) and vertical (**b**) activities in 10-min bins. Total distance travelled (**c**) and total rearing counts (**d**) during 120 min. Data presented as mean (n = 7-8) (**a** and **b**) or mean +/- SEM (n = 7-8) (**c** and **d**). *** $p < 0.001$; ** $p < 0.01$; * $p < 0.05$ against methamphetamine
