# Supplementary material for: Effects of the new generation α-pyrrolidinophenones on spontaneous locomotor activities in mice, and on extracellular dopamine and serotonin levels in the mouse striatum
Source: Forensic Toxicol. 2018 Feb 26;36(2):334–50. doi: 10.1007/s11419-018-0409-x (PMC6002449; doi:10.1007/s11419-018-0409-x)
Supplement: Supplementary file 3 — Supplementary material 3 (DOC 728 kb) [file 11419_2018_409_MOESM3_ESM.doc]

**Fig. S3** Presentation of typical probe placement (shaded bars) in the mouse striatum (according to Paxinos and Franklin 2008)
